# The morphology of the adrenal gland in the European bison (*Bison bonasus*)

**DOI:** 10.1186/s12917-016-0783-8

**Published:** 2016-08-03

**Authors:** Karolina Barszcz, Helena Przespolewska, Katarzyna Olbrych, Michał Czopowicz, Joanna Klećkowska-Nawrot, Karolina Goździewska-Harłajczuk, Marta Kupczyńska

**Affiliations:** 1 Department of Morphological Sciences, Faculty of Veterinary Medicine, Warsaw University of Life Sciences, 159 Nowoursynowska, 02-776 Warsaw, Poland; 2 Laboratory of Veterinary Epidemiology and Economics, Faculty of Veterinary Medicine, Warsaw University of Life Sciences, Nowoursynowska 159, 02-776 Warsaw, Poland; 3 Department of Animal Physiology and Biostructure, Faculty of Veterinary Medicine, Wroclaw University of Environmental and Life Sciences, Kozuchowska 1/3, 51-631 Wroclaw, Poland

**Keywords:** European bison, Adrenal gland, Morphology, Histology

## Abstract

**Background:**

The anatomy of the adrenal glands has been widely studied in many species of domestic and wild mammals. However, there are no available literature reports describing the morphology and morphometry of the adrenal glands of the European bison (*Bison bonasus*).

**Results:**

The study was conducted on 97 European bison of both sexes. The growth of the adrenal glands corresponded to the growth of the whole body, with the largest increase in size occurring in the first 2 years of the animal’s life, followed by a slower increase in size until the animal was 5–7 years old. There were no statistically significant differences between ipsilateral adrenal glands of males and females with respect to age. There was no statistically significant difference in weight between the left and the right adrenal gland. However, there was a difference in the length, width and thickness of the two glands. Reference intervals for adrenal gland size and weight were computed separately for two bison age groups (up to 2 years of age and older than two years). The adrenal gland consisted of a cortex and a medulla. The connective-tissue capsule contained two layers. It had a fibrous structure and abundant adipose tissue. The cortex was divided into three zones. The zona glomerulosa contained cells arranged in bundles and curves. Numerous apoptotic cells were observed among regular cells in the zona reticularis. There were vacuoles in the cells of both zona fasciculata and zona reticularis, which formed a foamy cytoplasm. The adrenal medulla was composed of large, dark cells with a highly basophilic cytoplasm in the superficial region and of smaller, lighter cells in deeper layers. Sinusoidal vessels were located in the central part of the medulla.

**Conclusions:**

The left adrenal gland was significantly longer, narrower and thinner than the right one. There were no significant differences in the structure of the adrenal medulla and cortex of the European bison compared to other species of domestic and wild mammals. There was a thick layer of adipose cells at numerous locations in the adrenal capsule of the bison.

**Electronic supplementary material:**

The online version of this article (doi:10.1186/s12917-016-0783-8) contains supplementary material, which is available to authorized users.

## Background

The European bison morphology has been studied since the 1920s. Although more than 100 original studies have been published focusing on the morphological structure of organ systems in the European bison [1, 2], few of them have analysed the endocrine glands in this species. Existing reports describe the morphology of the pancreas [3], thyroid gland [4–6] and thymus [7].

There are literature reports and figures showing the structure of the adrenal gland in various mammalian species, including domestic and wild animals. Studies on the adrenal gland have been carried out domestic animals: Nili-Ravi buffalo (*Bubalus bubalis*) [8], cattle [9], one-humped camel (*Camelus dromedarius*) [10–12], wild ruminants, such as in pampas deer (*Ozotoceros bezoarticus*) [13], as well as in other wild animals such as: mongoose (*Herpestes auropunctatus*) [14], common shrew (*Sorex araneus*), Eurasian pygmy shrew (*Sorex minutus*), muskrat (*Ondatra zibethicus*), bank vole (*Myodes glareolus*), field vole (*Microtus agrestis*) [15], bottlenose dolphin (*Tursiops truncatus*) [16, 17] and common seal (*Phoca vitulina vitulina*) [18]. However, there are no available studies on the morphology of the adrenal gland in the European bison.

The European bison is an endangered species. More information on its anatomy is warranted as there are few published studies of this species [1–7]. This study is the first report on the morphology of the adrenal gland in the European bison. Given the anatomical similarity between the European bison and other ruminants, the results of this study can be applied to other species, including the endangered ones.

The aim of this study was to describe the location, macroscopic and microscopic structure of the adrenal glands in the European bison, as well as to contribute to the current knowledge of comparative anatomy of wild mammals.

## Results

### Gross anatomy

**Fig. 1:**
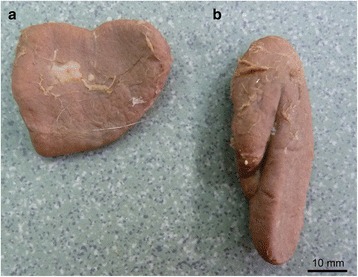
The adrenal gland in the European bison: **a** – right adrenal gland, **b** – left adrenal gland

Our study showed that the adrenal glands were positioned intraperitoneally in the European bison. The left adrenal gland was located distally from the caudal extremity of the left kidney at the level of L1. It lay behind the cranial mesenteric artery, very close to the medial plane. Its lateral border was adjacent to the rumen, and its medial border was positioned next to the caudal vena cava. In all the studied European bison, the shape of the left adrenal gland resembled that of number “1” (Fig. 1).

The right adrenal gland was situated at the level of Th13-Th14, in front of the cranial extremity of the right kidney. Its dorsal surface adhered to the right crus of the diaphragm. The ventral surface adhered to the renal impression on the liver, close to the caudal vena cava. The right adrenal gland was triangular in shape (Fig. 1).

### Statistical analysis

**Fig. 2:**
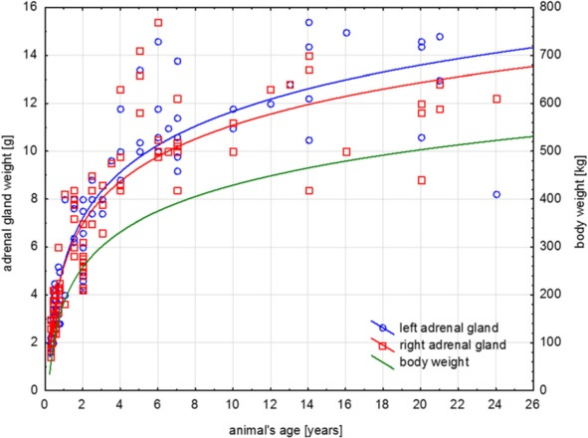
Growth of the left and the right adrenal gland with respect to the animal’s growth

The growth of the adrenal glands seemed to correspond to the growth of the whole body, with the largest increase in size occurring within the first two years of the animal’s life, followed by a slower increase in size until the animal was 5–7 years old (Fig. 2).

The weight and size of the adrenal glands are presented in Table 1. Taking into consideration the animals’ age, there were no statistically significant differences in the size and weight of the adrenal glands between males and females.

**Table 1:** Weight and size of the left and the right adrenal gland in males and females

	Left adrenal gland			Right adrenal gland		
	Female	Male	p ANCOVA	Female	Male	p ANCOVA
Length [cm]	54.03 ± 13.29	49.85 ± 11.19	0.360	39.60 ± 9.47	37.10 ± 8.90	0.262
Width [cm]	24.85 ± 5.60	21.75 ± 4.90	0.549	28.99 ± 7.45	27.03 ± 5.47	0.411
Thickness [cm]	9.47 ± 1.90	9.34 ± 1.79	0.186	11.42 ± 3.08	10.89 ± 2.37	0.286
Weight [g]	7.74 ± 4.34	5.84 ± 3.17	0.740	7.42 ± 3.97	5.76 ± 3.27	0.704
Relative weight	0.031 ± 0.009	0.032 ± 0.014	0.834	0.030 ± 0.010	0.032 ± 0.014	0.907

The change in the weight and size of the left and right adrenal glands in the three consecutive age groups is presented in Table 2. Due to the fact that there were no statistically significant differences between males and females, those groups included both sexes. There was a statistically significant overall difference among the age groups with respect to all the analysed parameters (p_ANOVA_ <0.001). The only exception was relative weight, which remained unchanged in all the age groups and only slightly declined in the older animals. However, that effect was not statistically significant. There were significant differences between the youngest and the adolescent European bison with respect to all the analysed parameters, whereas only some parameters differed significantly between the adolescent and the adult European bison. There was no difference between the weight of the left and the right adrenal gland within the age groups (*p* = 0.123). At the same time, all three size parameters differed between the left and the right gland. The left adrenal gland was significantly longer (*p* < 0.001) but narrower and thinner (both *p* < 0.001) than the right one in all the age groups. This corresponds to a different shape of the contralateral adrenal gland, as shown in the picture (Fig. 1).

**Table 2:** Weight and size of the left and the right adrenal gland in three age groups

Age group		Length [cm]	Width [cm]	Thickness [cm]	Weight [g]	Relative weight
		Left adrenal gland				
1	The youngest bison – up to 2 years-old (*n* = 58)	44.52 ± 8.91	19.92 ± 3.26	8.39 ± 1.43	4.16 ± 1.84	0.033 ± 0.013
2	Adolescent bison – 3–5 years-old (*n* = 14)	59.21 ± 4.19	26.70 ± 3.34	10.96 ± 1.54	9.31 ± 1.72	0.025 ± 0.005
3	Adult bison – older than 5 years (*n* = 25)	66.50 ± 7.11	30.29 ± 2.32	10.94 ± 1.11	12.14 ± 2.04	0.028 ± 0.005
	p Tukey test for unequal groups					
	class 1 vs. 2	<0.001^a^	<0.001^a^	<0.001^a^	<0.001^a^	0.090
	class 2 vs. 3	0.046^a^	0.007^a^	0.999	<0.001^a^	0.681
		Right adrenal gland				
1	The youngest bison – up to 2 years-old (*n* = 58)	32.82 ± 6.22	23.93 ± 4.34	9.75 ± 1.83	4.08 ± 1.73	0.034 ± 0.014
2	Adolescent bison – 3–5 years-old (*n* = 14)	45.56 ± 6.18	33.50 ± 3.52	12.28 ± 2.10	9.66 ± 2.34	0.026 ± 0.006
3	Adult bison – older than 5 years (*n* = 25)	48.00 ± 5.50	35.06 ± 4.55	13.94 ± 2.74	11.28 ± 1.80	0.027 ± 0.005
	p Tukey test for unequal groups					
	class 1 vs. 2	<0.001^a^	<0.001^a^	0.006^a^	<0.001^a^	0.191
	class 2 vs. 3	0.536	0.603	0.098	<0.001^a^	0.985

^a^Difference significant at α = 0.05

Ninety-five percent reference intervals (RI) for the adrenal gland size and weight are presented in Table 3, separately for the youngest and for the adolescent/adult bison. A common RI was used for the adolescent and the adult bison due to a small size of the sample groups and insignificant differences in their parameters.

**Table 3:** Reference intervals (RIs) of the weight and the size of the left and the right adrenal gland of the European bison

	Parameter	95 % reference interval (RI)					
		Youngest bison (*n* = 58)		Adolescent and adult bison (*n* = 39)		Overall (*n* = 97)	
		Lower limit (CI 90 %)	Upper limit (CI 90 %)	Lower limit (CI 90 %)	Upper limit (CI 90 %)	Lower limit (CI 90 %)	Upper limit (CI 90 %)
Left adrenal gland	Length [cm]	28.1 (25.9–32.3)	67.0 (59.1–69.5)	48.4 (46.4–52.5)	78.6 (75.1–81.6)	30.9 (25.9–33.6)	76.7 (72.1–81.5)
	Width [cm]	13.1 (11.5–15.4)	26.7 (25.6–27.0)	22.5 (21.1–24.4)	35.9 (34.6–37.6)	14.8 (11.5–16.0)	32.9 (32.2–35.5)
	Thickness [cm]	5.5 (5.5–6.1)	11.7 (10.5–12.1)	8.4 (7.8–9.1)	13.6 (13.1–14.3)	5.8 (5.5–6.6)	13.0 (12.3–13.1)
	Weight [g]	1.6 (1.6–2.0)	8.0 (7.8–8.0)	6.1 (5.4–7.1)	16.0 (14.8–17.0)	1.8 (1.6–2.1)	14.9 (14.4–15.4)
	Relative weight	0.019 (0.018–0.020)	0.083 (0.054–0.091)	0.017 (0.015–0.019)	0.038 (0.036–0.040)	0.019 (0.018–0.020)	0.067 (0.050–0.091)
Right adrenal gland	Length [cm]	23.1 (22.7–23.9)	47.4 (43.3–49.5)	34.8 (32.5–38.0)^a^	54.7 (57.3–61.9)	23.7 (22.7–24.9)	55.4 (54.0–56.0)
	Width [cm]	15.7 (15.5–17.1)	32.7 (31.0–33.2)	25.3 (23.2–27.9)	42.6 (40.4–45.0)	16.2 (15.5–18.3)	43.7 (38.2–46.2)
	Thickness [cm]	6.2 (6.0–7.0)	13.0 (12.4–13.0)	8.0 (6.9–9.1)	18.7 (17.5–19.9)	6.7 (6.0–7.0)	18.0 (16.5–21.1)
	Weight [g]	1.5 (1.4–2.0)	8.3 (7.6–8.4)	6.2 (5.4–7.1)	15.2 (14.1–16.2)	1.7 (1.4–2.2)	14.1 (13.3–15.4)
	Relative weight	0.017 (0.016–0.020)	0.089 (0.058–0.091)	0.015 (0.013–0.017)	0.037 (0.034–0.040)	0.018 (0.016–0.019)	0.076 (0.049–0.091)

The NPAR method was used to calculate all RIs in the youngest bison. The RUD method was used to calculate virtually all RIs in the adolescent and adult bison except for one case (^a^), in which the RTD method was used

### Histological analysis

**Fig. 3:**
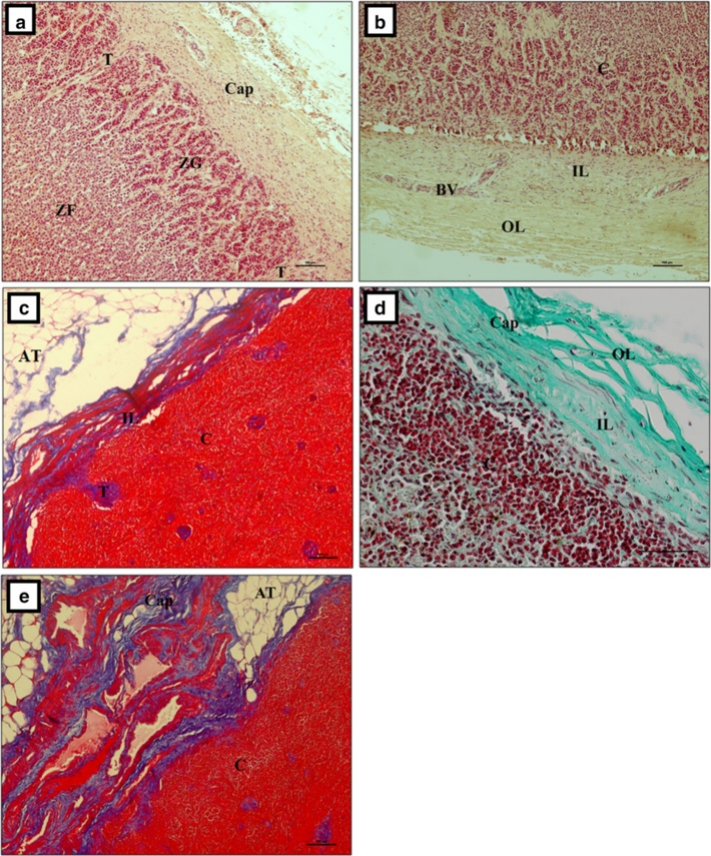
The adrenal gland in the European bison. **a** Thick connective tissue capsule, Stain = H&E, Bar = 100 μm; **b** The inner and outer layer of the connective tissue capsule, Stain = H&E, Bar = 100 μm; **c** Adipose and inner layer of connective tissue capsule formed from collagen fibers, Stain = Azan trichrome, Bar = 100 μm; **d** Inner layer of connective tissue capsule with thick and regular strands of elastic fibers. Stain = Masson-Goldner trichrome. Bar = 50 μm; **e** Collagen fibers arranged in a mesh. Stain = Azan trichrome. Bar = 100 μm. Cap – capsule, OL – outer layer, IL – inner layer, AT – adipose tissue, T – trabeculae, ZG – zona glomerulosa, ZF – zona fasciculata, C – cortex, BV – blood vessels

**Fig. 4:**
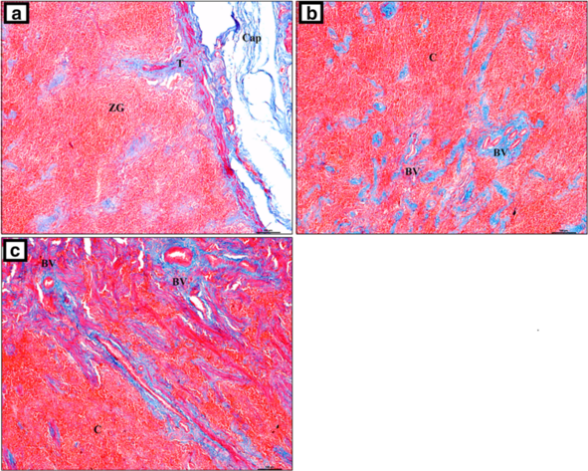
The adrenal gland in the European bison. Stain = Azan trichrome. Bar = 100 μm. **a** Inner layer of the connective tissue layer forming thick connective tissue trabeculae that penetrate the zona glomerulosa; **b** Numerous blood vessels in the adrenal cortex; **c** Numerous blood vessels surrounded by collagen fibers forming thick circular layers in the adrenal cortex. Cap – capsule, T – trabecule, ZG – zona glomerulosa, C – cortex, BV – blood vessels

Our study showed that the adrenal gland of the European bison was composed of the cortex and medulla. It was covered by a thick connective tissue capsule (Fig. 3a), which had two layers (Fig. 3b). The outer layer was formed from collagen, elastin and smooth muscle fibers (Fig. 3c and d). Abundant adipose tissue (Fig. 3c and e) was observed between the inner and the outer layer. Sometimes, those fibers formed small irregular clusters. The inner layer was formed from tightly aligned collagen fibers (Fig. 3d). Looser connective tissue and an extensive network of well-developed blood vessels were found on the border of those two layers (Fig. 3b). The internal layer of the capsule formed strands that penetrated the cortex (Fig. 3a). In turn, the cortex was divided into *zona glomerulosa s. zona arcuata*, *zona fasciculata* and *zona reticularis*. Collagen fibers formed numerous, irregular, thin strands in the zona glomerulosa that ran between cells. They formed large and thick trabeculae, which divided the zona glomerulosa and penetrated deep into the organ (Fig. 4a). The collagen fibers formed irregularly distributed clusters between cells in the deeper layers of the adrenal gland (Fig. 4b). Numerous blood vessels were observed at that level (Fig. 4c). Collagen fibers were arranged in thick circular layers around them (Fig. 4c).

**Fig. 5:**
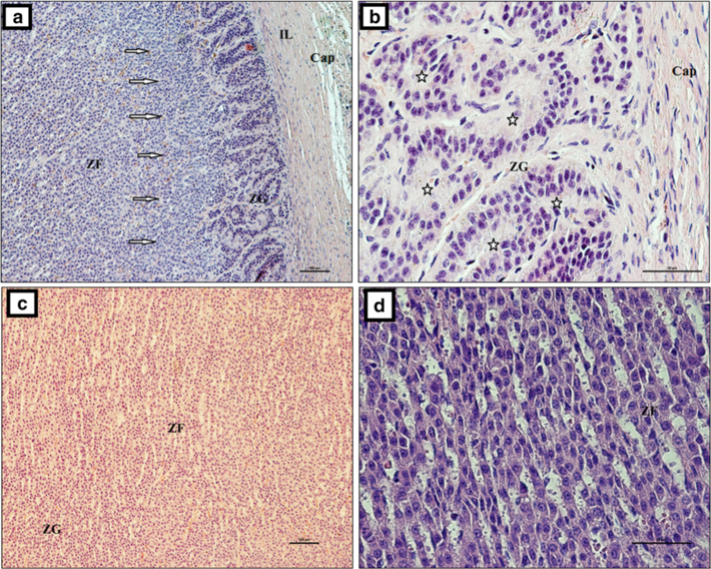
The adrenal gland in the European bison. Stain = H&E. **a** Glandular cells of the zona glomerulosa are arranged in the form of bundles and arches (white arrow), Bar = 100 μm; **b** A concentric arrangement of the cells of the zona glomerulosa (white star), Bar = 50 μm; **c** Arches that form columns creating the zona fasciculata, Bar = 50 μm; **d** Oval cells are arranged in strands of arches, Bar = 50 μm. Cap – capsule, IL – inner layer, ZG – zona glomerulosa, ZF – zona fasciculata

**Fig. 6:**
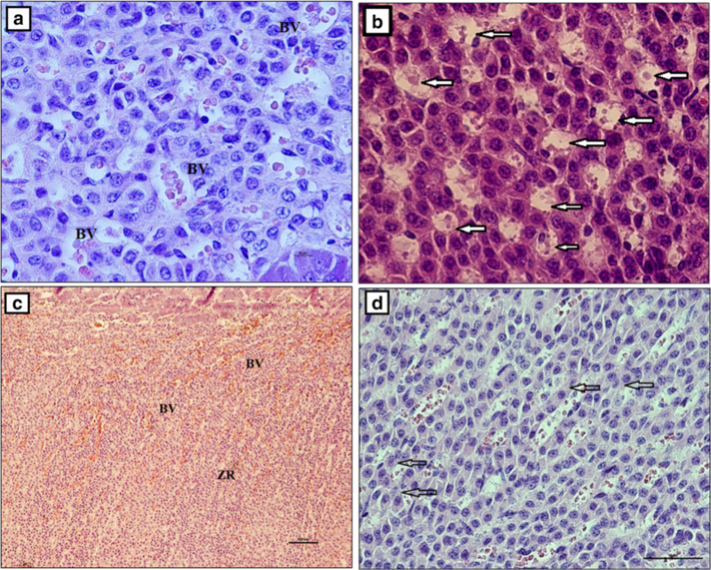
The adrenal gland in the European bison. Stain = H&E. **a** Cells of the zona reticulata of various shape and irregularly arranged form a network, Bar = 10 μm; **b** Numerous apoptotic cells (white arrow), Bar = 10 μm; **c** Blood-filled vessels, Bar = 100 μm; **d** Cells of the zona fasciculata with vacuoles (black arrow), Bar = 50 μm. ZR – zona reticularis, BV – blood vessels

Glandular cells of the zona glomerulosa formed bundles and arches (Fig. 5a). The bundles were located beneath the capsule and consisted of tightly and concentrically arranged cells. The cells were polygonal in shape (Fig. 5b). In contrast, the arches formed clear columns that penetrated the gland, forming a thin zona fasiculata (Fig. 5c). The cells of those structures were oval and were arranged in single or multiple arch-shaped strands (Fig. 5d). Connective tissue and numerous blood vessels were present in both layers of the adrenal cortex. The zona reticularis was prominent and wide, and it was formed by irregularly arranged cells of various shapes (cubic, fusiform, oval, polygonal) (Fig. 6a). There were numerous apoptotic cells in the zona reticularis (Fig. 6b). That layer also contained numerous vessels (Fig. 6c). Cells with vacuoles formed a foamy cytoplasm in the zona fasciculata and zona reticularis (Fig. 6d).

**Fig. 7:**
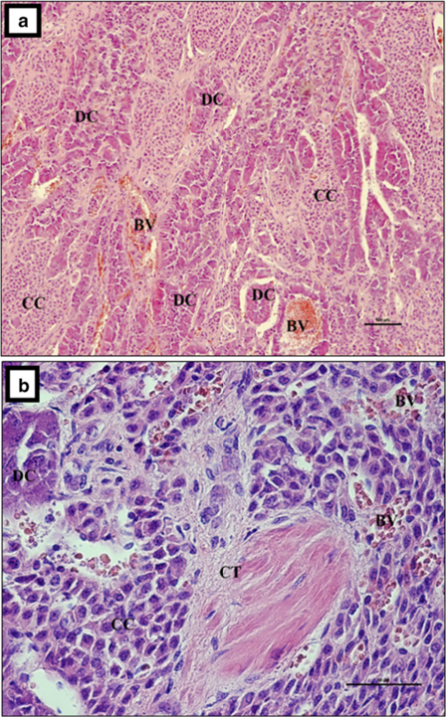
The adrenal gland in European bison. Stain = H&E. **a** The bigger, dark cells and the smaller light cells of the adrenal medulla are visible, Bar = 100 μm; **b** The bigger, dark cells and the smaller light cells of the adrenal medulla are visible, Bar = 50 μm. DC – dark cells, CC – clear cells, CT – connective tissue, BV – blood vessels

**Fig. 8:**
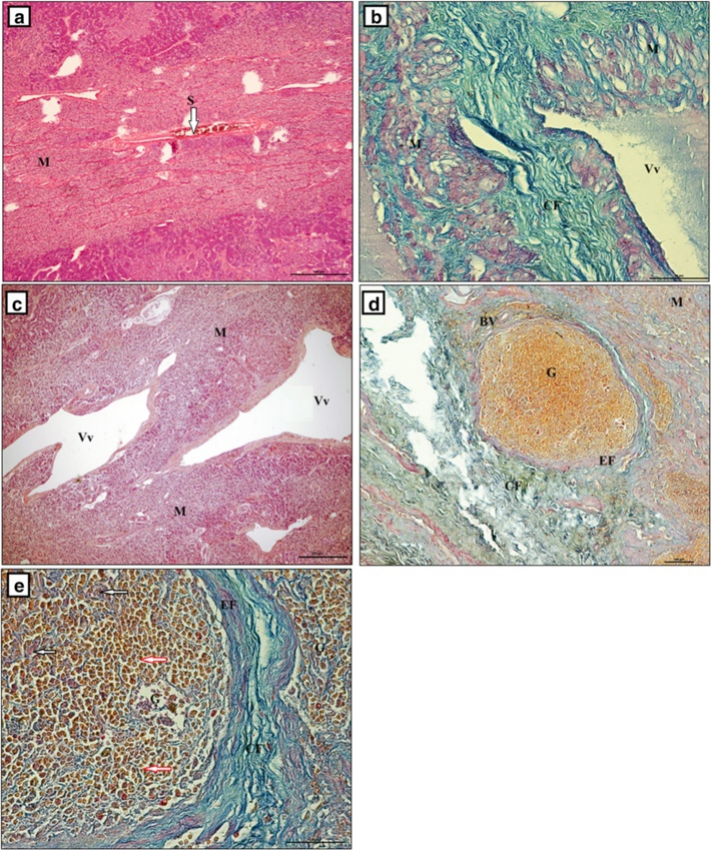
The adrenal gland in the European bison. **a** Single sinusoidal vessels in the central part of the adrenal medulla, Stain = H&E, Bar = 100 μm; **b** Veins surrounded by loose connective tissue, Stain = Masson-Goldner trichrome, Bar = 50 μm; **c** Veins in the adrenal medulla, Stain = H&E, Bar = 50 μm; **d** Nerve ganglion and vein in the adrenal medulla, Stain = Mallory trichrome. Bar = 100 μm; **e** Nerve ganglion in adrenal medulla, Stain = Mallory trichrome. Bar = 50 μm. M – medulla, G – ganglion, CF- collagenous fibres, EF – elastic fibres, black arrow – large polygonal cells stained pink, red arrow – polygonal cells stained light - or dark orange, BV – blood vessels, EF – elastic fibres, Vv – Venous vessels, S – sinusoid

The adrenal medulla consisted of larger dark cells and smaller, light ones (Fig. 7a). The dark cells were concentrated and formed elongated, dense and nest-like shapes. Those nest-like structures were situated directly beneath the adrenal cortex and had a ring-like distribution surrounding the medulla. The medulla was formed from light cells (Fig. 7a). The dark cells had an irregular, elongated shape and basophilic cytoplasm. The borders between those cells were ill-defined (Fig. 7b), and there were single, large and thick vessels between them (Fig. 7a). Light cells were located deeper within the adrenal medulla and were arranged in rows. They had a light, weakly basophilic, foamy cytoplasm (Fig. 7b). There were blood vessels of various diameters located between them, while sinusoidal vessels (single, double or triple) were located in the central part of the medulla (Fig. 8a). Additionally, numerous ganglion cells were present. They were situated between the light cells and formed oval plexuses surrounded by loose connective tissue and veins (Fig. 8b, c and d). Single, large pink polygonal multipolar nerve cells were seen within the plexus. Light or dark-orange polygonal ganglion cells predominated and formed numerous oval shaped clusters (Fig. 8e).

## Discussion

Numerous studies on the shape of the adrenal gland were carried out in various wild and domesticated animal species [9–11, 14–16, 19, 20]. The shape of the adrenal glands may differ not only between species but also between animals of the same species. In all the examined European bison, the left adrenal gland resembled the shape of number “1”, while the right adrenal gland was triangular. A similar adrenal shape was described in domestic cattle and small ruminants [19]. Studies carried out in the Nili-Ravi buffalo showed that the left adrenal gland had a V-shape, while the right gland was C-shaped [8].

Erdoğan and Pérez [13] described the shape of the adrenal glands in the pampas deer and found inter-individual differences in the shape of the left gland. Two of the ten studied deer had a V- or heart-shaped left gland, while the remaining eight animals had an oval shaped one. The right gland was oval-shaped in all the studied deer.

There are several morphometric studies of the adrenal gland in literature. They describe the relative and absolute weight, length, width, thickness and circumference of each gland. However, our study is the first to report reference intervals for size and weight of the adrenal glands which may prove useful to clinicians, in particular if abdominal sonography is planned as a diagnostic method in sedated European bison.

The studies carried out in the pampas deer by Erdoğan and Pérez [13] revealed that the right adrenal gland mean weight was 0.59 ± 0.09 g, and the mean weight of the left adrenal gland was 0.64 ± 0.1 g. The mean length and width of the left adrenal gland (18.04 ± 0.94 mm, 11.83 ± 0.81 mm) was larger than the right gland (17.3 ± 1.58 mm, 10.11 ± 0.42 mm). The mean thickness of the right adrenal gland (5.11 ± 0.45 mm) was greater than that of the left gland (4.59 ± 0.31 mm).

Hussain et al. [8] presented the absolute and relative weight of the adrenal glands in the Nili-Ravi buffalo. The absolute weight was 23.70 ± 1.12 g, and the relative weight was 0.06 ± 0.001 % in young animals. In old animals, the absolute and relative weights were 33.85 ± 1.17 g and 0.05 ± 0.001 g, respectively. The authors provided the length and width of both the left and the right adrenal glands. The length and width of the right adrenal gland in young animals were 5.03 ± 0.05 cm and 3.84 ± 0.06 cm, and 6.69 ± 0.09 cm and 4.13 ± 0.0015 cm in adult animals, respectively. In the left adrenal gland, those measurements were 5.17 ± 0.08 cm and 2.85 ± 0.05 cm in young animals and 6.85 ± 0.100 cm and 4.82 ± 0.22 cm in adult animals, respectively. The mean length of the adrenal glands was significantly greater in adults than in young individuals. However, the difference in the width of the right adrenal gland between young and adult buffaloes was non-significant [8].

In the studied European bison population, there was no difference in the weight of adrenal glands with respect to age. However, all three size parameters differed between the left and the right gland. The left adrenal gland was significantly longer, narrower and thinner than the right one in all the age groups.

In the European bison, like in other mammalian species, the capsule consisted of an inner and outer layer, which differed in the amount of connective tissue [19–21]. Interestingly, we found a substantial layer of adipose cells between the inner and the outer layer of the capsule, which may be presumed to be characteristic of wild animals. There are no literature reports describing a similar finding in domestic animals. According to Nabipour [22], the adrenal capsule in camels (*Camelus dromedarius*) has dense irregular connective tissue. Al-Bagdadi [10] reported that the adrenal capsule in camels (*Camelus dromedarius*) consists of two layers, and the inner layer contains cellular elements. Similarly to domestic animals, the adrenal capsule in the European bison contained a lot of collagen, elastin and smooth muscle fibers [19–21]. Vuković et al. [17] found mainly collagen fibres and a few elastin fibres. In the European bison, similarly to domestic animals and camels, the adrenal capsule was well developed and formed connective tissue trabeculae that penetrated the cortex and rarely entered the medulla [10, 19–22]. In contrast, Vuković et al. [17] reported that in marine mammals (dolphins) the thick connective tissue trabeculae did not divide the cortex into pseudo-lobes.

Similarly to other land and marine mammals, the adrenal gland in the European bison consisted of the cortex and medulla. The cortex contained three layers – the zona glomerulosa, zona fasciculata and zona reticularis.

In the zona glomerulosa of the European bison, the cells formed numerous bundles and arches, similarly to domestic ruminants, horse and carnivores [19–21, 23]. The cells of the zona glomerulosa in the European bison were polygonal in shape as in domestic mammals, camels and marine mammals [9, 10, 22].

In the European bison, as in humans and domestic ruminants, the cells in the zona fasciculata were predominantly arranged in bundles [24]. They were oval and arranged in single or multiple arch-shaped strands. On the other hand, in the zona fasciculata of the marine mammals (dolphin), the arches passed into columns and contained one or two cell rows in each cord [17]. Those cells were large and polygonal in marine mammals. Our research showed numerous apoptotic cells in the zona reticularis. A similar finding was described in cattle [9]. In the European bison and camel (*Camelus dromedaries*), many cells in the zona fasciculata and zona reticulata contained vacuoles, which formed a foamy cytoplasm and indicated the presence of lipids [12]. In the European bison, the structure of the adrenal medulla was similar to that of domestic animals [9, 19–21]. In the superficial layer of the adrenal medulla, there were large dark cells with a strongly basophilic cytoplasm. Small, lighter cells were present in the deeper layer of the medulla. According to Jelinek and Konecny [9], both types of cells were intertwined in cattle. According to Dellman [21], the medulla was divided into two parts in domestic animals (horses, cows, sheep and pigs). The outer part was composed of strongly staining epinephrine-secretin cells, while the inner part was formed from cells with a weaker stain affinity (norepinephrine-secreting cells). A similar structure of the adrenal medulla was reported in marine mammals by Vuković et al. [17].

## Conclusions

The left adrenal gland in the European bison was significantly longer, narrower and thinner than the right one in all the age groups. There were no significant differences in the structure of the adrenal medulla and cortex of the European bison compared to other species of domestic and wild mammals. There was a thick layer of adipose cells at numerous locations in the adrenal capsule, which had not been described in land and marine mammals.

## Methods

### Animals and gross anatomy

The study material comprised adrenal glands from the 97 European bison (*Bison bonasus*) of both sexes (40 males and 57 females), aged 3 months to 24 years (median of 24 months, IQR from 7 to 72 months), inhabiting the Białowieza Forest (Białowieza National Park, Poland) (Additional file 1). The females were significantly older than the males (*p* = 0.006) with a median (IQR) of 24 (7–84) and 18 (5–30) months, respectively.

The animals were divided into three groups depending on their age: 3 months to 2 years old (group I), 2 to 5 years old (group II), and older than 5 years (group III). The animals were also classified based on data on their growth and development [25–27]. The information on the animals’ age was drawn from the Białowieza Nature Reserve Record Book.

The weight of each animal was determined under field conditions after legal culls were performed. After the abdominal cavity was opened, the holotopy, skeletotopy and syntopy of the adrenal glands were recorded. The adrenal glands were then removed, and the shape and mass of the left and the right gland were recorded using the AXIS AD2000 laboratory weighing scale, accurate to the nearest 0.01 g. Next, an electronic (TESA – CAL IP67) caliper, accurate to the nearest 0.01 mm, was used to measure the length, width and thickness of each gland.

The Bialowieza National Park was responsible for the culling of the European bison. The animals were not killed for the purpose of this study. Population control, bone fractures and car accidents were the most common reasons for culling the animals. The culling was carried out with the permission of the Ministry of Environment and the General Director for Environmental Protection in Poland (decision number: DOP-OZGIZ. 6401.06.7.2012.ls, DOP-OZ.6401.06.7.2012.ls.1 and DLP-III-4102-459/36490/14/ZK). The adrenal glands and other organs were collected by the authors (veterinarians) during dissection. The autopsy protocols are available in Bialowieza National Park. According to the Polish law, tests on tissues obtained *post-mortem* do not require an approval of the Ethics Committee (Parliament of the Republic of Poland, 1997).

The terminology used in the manuscript is in accordance with prevailing veterinary nomenclature [28].

### Statistical analysis

Numerical variables are presented as mean ± standard deviation (SD) except for the animal age, which is presented as the median and interquartile range (IQR). A Mann-Whitney U test was used to compare the age of the males and females. Since there was a significant difference in age between sexes, the female and male adrenal gland mass and size were compared, using the analysis of covariance (ANCOVA), with the animals’ age as a covariate. One-way analysis of variance (ANOVA) followed by the Tukey post-hoc test for unequal groups were used to compare the adrenal gland mass and size in the three age groups. ANOVA for paired samples was applied to compare the weight and size of the left and the right adrenal glands. For all the statistical tests, a significance level (α) of 0.05 was assumed. The analyses were performed using Statistica 10 software (StatSoft Inc.).

A 95 % reference intervals (RI) together with their 90 % confidence intervals (CI 90 %) were computed for adrenal gland weight and size (i.e. length, width and height) using either a nonparametric (NPAR) or robust method with or without Box-Cox transformation (RTD and RUD, respectively) depending on the results of the Anderson-Darling normality test and symmetry test. Given the small number of animals in the adolescent and adult bison groups (14 and 25, respectively), those classes were merged into one category (*n* = 39) for the needs of reference interval calculation. Moreover, the overall reference intervals were computed for all 97 bison. All the calculations of RIs were performed using the Reference Value Advisor [29].

### Histological study

The research material was directly fixed in 4 % buffered formaldehyde for 72 h, rinsed in running water for 24 h, processed in a vacuum tissue processor – ETP (RVG3, INTELSINT, Italy), embedded in paraffin and cut on a Slide 2003 (Pfm A.g., Germany) sliding microtome into 3–4 μm sections. The samples were then stained with haematoxylin and eosin (H&E), Azan trichrome, Masson-Goldner trichrome and Mallory trichrome to observe the general structure of the adrenal glands. The H&E, Azan trichrome, Masson-Goldner and Mallory trichrome staining scoring system was based on a standard protocol previously described [30]. All the obtained slides were examined using the Nikon Eclipse 80i light microscope (NIKON, Tokyo, Japan) for histological description.

## Additional file

Additional file 1: Measurements of the adrenal glands in the studied European bison population. (DOC 275 kb)
