# Supplementary material for: The morphology of the adrenal gland in the European bison (Bison bonasus)
Source: BMC Vet Res. 2016 Aug 3;12:161. doi: 10.1186/s12917-016-0783-8 (PMC4972984; doi:10.1186/s12917-016-0783-8)
Supplement: Additional file 1: — Measurements of the adrenal glands in the studied European bison population. (DOC 275 kb) [file 12917_2016_783_MOESM1_ESM.doc]

| Lp. | Sex | Age  (months) | Bodyweight  (kg) | Left adrenal gland | | | | Right adrenal gland | | | |
| --- | --- | --- | --- | --- | --- | --- | --- | --- | --- | --- | --- |
|  |  |  |  | weight  (g) | widht  (mm) | length  (mm) | thickness  (mm) | weight  (g) | width  (mm) | length  (mm) | thickness  (mm) |
| 1 | f | 3 | 38 | 1,6 | 14,9 | 25,9 | 6,2 | 1,4 | 18 | 25 | 8 |
| 2 | f | 3 | 30 | 2,2 | 18,1 | 30,5 | 7 | 2,6 | 18,6 | 26,6 | 9 |
| 3 | f | 4 | 72 | 2,4 | 17,5 | 34,8 | 7 | 2 | 19 | 23,6 | 7 |
| 4 | f | 4 | 86 | 2,2 | 16,3 | 33,2 | 8 | 2,2 | 22,7 | 28 | 6 |
| 5 | f | 5 | 100 | 3,6 | 22,4 | 44,5 | 6,1 | 3,5 | 23 | 30 | 6,5 |
| 6 | f | 5 | 70 | 2 | 14,8 | 41,4 | 5,6 | 2,6 | 21,9 | 33,4 | 7 |
| 7 | f | 5 | - | 3,4 | 17,4 | 38,1 | 9 | 3,4 | 22 | 26,6 | 9 |
| 8 | f | 6 | 100 | 3,2 | 17,5 | 41,2 | 7,5 | 2,8 | 23,2 | 30,4 | 8,1 |
| 9 | f | 6 | 110 | 4 | 20 | 46,2 | 9 | 3,8 | 22,5 | 34,8 | 10 |
| 10 | f | 6 | 100 | 4,5 | 21 | 45,5 | 9,5 | 4,2 | 24,4 | 30,5 | 10,5 |
| 11 | f | 6 | 108 | 2,8 | 17,8 | 36,6 | 8 | 2,8 | 26,9 | 27,7 | 7 |
| 12 | f | 7 | 80 | 3 | 19,9 | 38,6 | 6,4 | 3 | 21,3 | 28,2 | 7 |
| 13 | f | 7 | 105 | 3,4 | 27 | 42,5 | 9 | 2,8 | 17,8 | 32,6 | 11,7 |
| 14 | f | 7 | 96 | 2,8 | 18,7 | 33,8 | 9 | 2,6 | 15,5 | 30 | 10 |
| 15 | f | 7 | - | 3,8 | 19,7 | 43,9 | 8,7 | 2,9 | 16 | 33 | 12 |
| 16 | f | 8 | 125 | 3,2 | 21,2 | 38,1 | 8,1 | 3,4 | 19,6 | 30,3 | 12,2 |
| 17 | f | 8 | 110 | 5,2 | 21,5 | 48,5 | 10,3 | 6 | 17 | 31 | 11,5 |
| 18 | f | 9 | 140 | 2,8 | 17,6 | 43,8 | 7,2 | 4,5 | 16,5 | 29,5 | 12 |
| 19 | f | 9 | 146 | 3,8 | 18,6 | 46,9 | 8,2 | 3,8 | 25 | 30,5 | 10,5 |
| 20 | f | 12 | 280 | 8 | 24,8 | 61 | 7,6 | 8,2 | 31,6 | 45,1 | 10,5 |
| 21 | f | 12 | 220 | 4 | 21,2 | 47,4 | 9 | 3,6 | 23,3 | 33,9 | 7 |
| 22 | f | 18 | 180 | 7,6 | 26,4 | 52 | 10,1 | 7,2 | 32,1 | 40,4 | 10,2 |
| 23 | f | 24 | 197 | 4,2 | 20,3 | 46,5 | 9,8 | 5,5 | 30,5 | 40,5 | 9,5 |
| 24 | f | 24 | 243 | 4,8 | 21,9 | 45,1 | 7,9 | 4,8 | 29,5 | 34,6 | 10 |
| 25 | f | 24 | 190 | 4,4 | 26,2 | 47 | 5,5 | 4,2 | 25,7 | 32,1 | 8,2 |
| 26 | f | 24 | 240 | 4,8 | 20,5 | 51,6 | 7,4 | 4,3 | 27 | 32,5 | 9 |
| 27 | f | 24 | 193 | 5,4 | 22,5 | 49,8 | 8,4 | 5,2 | 25,6 | 30,8 | 11,2 |
| 28 | f | 24 | 230 | 5 | 22,5 | 46,2 | 9,2 | 5,2 | 30,4 | 35,7 | 11,1 |
| 29 | f | 24 | 290 | 6,6 | 18,4 | 69,5 | 8 | 7 | 28,6 | 49,5 | 10,5 |
| 30 | f | 30 | 270 | 8 | 26,3 | 60,5 | 10,4 | 9 | 29 | 41 | 10 |
| 31 | f | 36 | 250 | 7,4 | 22,4 | 58,5 | 10,6 | 6,6 | 29,7 | 35,3 | 10,1 |
| 32 | f | 48 | 458 | 10 | 31,9 | 54,8 | 12 | 9,8 | 33 | 46 | 11,7 |
| 33 | f | 48 | 430 | 8,8 | 26,5 | 59 | 10,5 | 8,4 | 32,6 | 38,8 | 12,1 |
| 34 | f | 60 | 420 | 13,4 | 26,4 | 54,3 | 13,1 | 14,2 | 39,2 | 52,1 | 13,6 |
| 35 | f | 60 | 310 | 10,4 | 27,6 | 57 | 10 | 11,6 | 34,2 | 54,5 | 14,1 |
| 36 | f | 72 | 442 | 11,8 | 29 | 61,6 | 11,2 | 10,5 | 35 | 43 | 12,5 |
| 37 | f | 72 | 430 | 10 | 30 | 62 | 11,5 | 9,8 | 32,3 | 42,5 | 13,2 |
| 38 | f | 72 | 470 | 14,6 | 32,2 | 78,6 | 10,4 | 15,4 | 46,2 | 55,8 | 17,9 |
| 39 | f | 78 | 410 | 11 | 28,9 | 54,9 | 11,9 | 10 | 34 | 41 | 13,5 |
| 40 | f | 84 | 360 | 10,6 | 30,3 | 68,5 | 11 | 10,2 | 32,5 | 54 | 12 |
| 41 | f | 84 | 400 | 11,4 | 29,1 | 61,7 | 11 | 10,4 | 34,5 | 43 | 12,1 |
| 42 | f | 84 | 380 | 9,2 | 27,1 | 56 | 11,9 | 8,4 | 35,5 | 37,4 | 15,2 |
| 43 | f | 84 | 400 | 13,8 | 29,6 | 69,2 | 12,4 | 12,2 | 33,6 | 56 | 15,2 |
| 44 | f | 120 | 390 | 11 | 30,3 | 61,2 | 12,1 | 10 | 32 | 42,5 | 15 |
| 45 | f | 120 | 420 | 11,8 | 29 | 67,9 | 11,2 | 11,2 | 44,8 | 52,2 | 15 |
| 46 | f | 144 | 400 | 12 | 30,8 | 73,6 | 9,8 | 12,6 | 35,2 | 47,5 | 17,6 |
| 47 | f | 156 | 380 | 12,8 | 30 | 70,5 | 9 | 12,8 | 36 | 47,5 | 11,1 |
| 48 | f | 168 | 400 | 10,5 | 31 | 57 | 10,1 | 8,4 | 30 | 46,1 | 11,7 |
| 49 | f | 168 | 340 | 12,2 | 32 | 71 | 10 | 13,4 | 40,1 | 53,2 | 14,2 |
| 50 | f | 168 | 440 | 15,4 | 33,2 | 71,7 | 11,9 | 13,4 | 30,5 | 49,9 | 16,5 |
| 51 | f | 192 | 480 | 15 | 32 | 68 | 12 | 10 | 34,3 | 48,9 | 12,4 |
| 52 | f | 240 | 460 | 14,6 | 31,2 | 67 | 10,7 | 11,6 | 36,4 | 51,1 | 12,1 |
| 53 | f | 240 | 420 | 14,4 | 32,5 | 81,5 | 10,6 | 12 | 34 | 50 | 12 |
| 54 | f | 240 | 420 | 10,6 | 28,1 | 61,1 | 11,9 | 8,8 | 29,1 | 38 | 15,7 |
| 55 | f | 252 | 430 | 13 | 32 | 71 | 10 | 12,8 | 42,4 | 50,5 | 9,1 |
| 56 | f | 252 | 410 | 14,8 | 35,5 | 74,3 | 11,7 | 11,8 | 36 | 50 | 12 |
| 57 | f | 288 | 370 | 8,2 | 24,8 | 57,3 | 8,1 | 12,2 | 35 | 52,7 | 21,1 |
| 58 | m | 3 | 33 | 3 | 18 | 36,5 | 10,6 | 3 | 21,4 | 28,5 | 9 |
| 59 | m | 3 | 66 | 2 | 15,3 | 31,4 | 8,2 | 1,8 | 19,4 | 22,7 | 8,4 |
| 60 | m | 3 | 46 | 1,6 | 11,5 | 33,5 | 7,4 | 1,7 | 19 | 24 | 8,5 |
| 61 | m | 4 | 35 | 2 | 17,1 | 34,8 | 7 | 2,2 | 21,2 | 26,1 | 8 |
| 62 | m | 5 | 72 | 4,2 | 19,6 | 43 | 10 | 4,2 | 27 | 34,2 | 11,5 |
| 63 | m | 5 | 70 | 2,4 | 17,4 | 34,6 | 8 | 2,6 | 20,2 | 23,8 | 9 |
| 64 | m | 5 | 108 | 2,6 | 19,5 | 37,8 | 8,2 | 2,5 | 20 | 24 | 9,5 |
| 65 | m | 5 | 97 | 2 | 17,7 | 32,7 | 6 | 2,4 | 21,6 | 26,6 | 8 |
| 66 | m | 5 | 100 | 2,6 | 16,3 | 39,4 | 8 | 2,8 | 24,9 | 31,1 | 7 |
| 67 | m | 5 | 78 | 3 | 20,1 | 40 | 7,7 | 2,4 | 24,5 | 29,4 | 9,2 |
| 68 | m | 5 | 85 | 3,6 | 17,6 | 46,7 | 7 | 3,2 | 25,4 | 31,5 | 9,5 |
| 69 | m | 5 | 88 | 4,2 | 19,3 | 47,7 | 8,6 | 3,8 | 23,5 | 35,6 | 10,3 |
| 70 | m | 7 | 102 | 4,2 | 20,7 | 46,8 | 7 | 4 | 25,4 | 34,1 | 9 |
| 71 | m | 7 | 91 | 2,6 | 16,3 | 41,3 | 7 | 2,4 | 19,1 | 29,1 | 8 |
| 72 | m | 7 | - | 3,2 | 20,1 | 41,9 | 8 | 3,2 | 25 | 30,2 | 12,2 |
| 73 | m | 8 | 130 | 3,2 | 16 | 37,1 | 11,3 | 3,3 | 24 | 29 | 11,5 |
| 74 | m | 8 | 100 | 3,6 | 18,1 | 44,2 | 10,2 | 4,2 | 25,8 | 29,5 | 11,4 |
| 75 | m | 8 | - | 2,8 | 16,1 | 38,3 | 8,5 | 4,3 | 26 | 29 | 9 |
| 76 | m | 9 | 100 | 5 | 21,3 | 49,3 | 10,4 | 4,2 | 27,5 | 38,2 | 12,9 |
| 77 | m | 18 | 250 | 6,2 | 26,1 | 47 | 9,1 | 6 | 29,8 | 43,7 | 10 |
| 78 | m | 18 | 190 | 7,8 | 22,2 | 53,8 | 12,1 | 8,4 | 30,9 | 41,5 | 12,9 |
| 79 | m | 18 | 210 | 7,6 | 24,6 | 54 | 10,1 | 8 | 31 | 42 | 12,5 |
| 80 | m | 18 | 230 | 6,4 | 23,3 | 55,3 | 10,5 | 5,6 | 26,2 | 40 | 13 |
| 81 | m | 18 | 190 | 8 | 25,1 | 57,1 | 9,3 | 7,8 | 33,2 | 42,2 | 12,1 |
| 82 | m | 24 | 302 | 7,5 | 24 | 56 | 9 | 5,6 | 26,2 | 39,5 | 11,5 |
| 83 | m | 24 | 250 | 6,6 | 22,5 | 64,3 | 8,2 | 5,4 | 23,6 | 44,2 | 9,6 |
| 84 | m | 24 | 200 | 6 | 20,5 | 51,5 | 9,6 | 4,8 | 24,5 | 35 | 9,2 |
| 85 | m | 24 | 300 | 8 | 22,7 | 62 | 9,3 | 6,2 | 23,5 | 42 | 11,7 |
| 86 | m | 24 | 240 | 4,6 | 19,5 | 54,1 | 7,4 | 5 | 23 | 40 | 9 |
| 87 | m | 30 | 300 | 8,8 | 24,3 | 59,6 | 13 | 8,4 | 32,5 | 49,1 | 14,1 |
| 88 | m | 30 | 290 | 7,4 | 22,5 | 62,4 | 9,6 | 7 | 28,1 | 42,3 | 11,7 |
| 89 | m | 36 | 320 | 8 | 26,2 | 53,6 | 8,5 | 7,8 | 29,5 | 40 | 9 |
| 90 | m | 36 | 390 | 8 | 27 | 54 | 9 | 8,6 | 37,1 | 41,8 | 12,4 |
| 91 | m | 42 | 430 | 9,6 | 22,2 | 63,8 | 11,2 | 9,5 | 37 | 42 | 12 |
| 92 | m | 48 | 450 | 8,8 | 26,7 | 67,8 | 10 | 8,6 | 36,9 | 49,8 | 10,1 |
| 93 | m | 48 | 530 | 11,8 | 31,8 | 61,7 | 12,5 | 12,6 | 36,6 | 51,2 | 14,5 |
| 94 | m | 60 | 470 | 10 | 32 | 62 | 13 | 13,2 | 33,6 | 54 | 16,5 |
| 95 | m | 72 | 460 | 10,6 | 26,1 | 62,8 | 12 | 10 | 26,1 | 41,8 | 18 |
| 96 | m | 84 | 490 | 9,8 | 32,5 | 61 | 9,4 | 10 | 34 | 50,5 | 11,5 |
| 97 | m | 168 | 740 | 14,4 | 30 | 73 | 11,7 | 14 | 37 | 55 | 12,5 |
